# Further Evidence That Cannabis Moderates Familial Correlation of Psychosis-Related Experiences

**DOI:** 10.1371/journal.pone.0137625

**Published:** 2015-09-18

**Authors:** Ruud van Winkel, GROUP Investigators

**Affiliations:** 1 University Psychiatric Center KU, Leuven, Belgium; 2 Maastricht University Medical Centre, South Limburg Mental Health Research and Teaching Network, EURON, Maastricht, The Netherlands; Macquarie University, AUSTRALIA

## Abstract

**Background:**

Familial correlations underlie heritability estimates of psychosis. If gene-environment interactions are important, familial correlation will vary as a function of environmental exposure.

**Methods:**

Associations between sibling and parental schizotypy (n = 669 pairs, n = 1222 observations), and between sibling schizotypy and patient CAPE psychosis (n = 978 pairs, n = 1723 observations) were examined as a function of sibling cannabis use. This design is based on the prediction that in unaffected siblings who are not exposed, vulnerability for psychosis will remain latent, whereas in case of exposure, latent psychosis vulnerability may become expressed, at the level of schizotypal symptoms, causing the phenotypic correlation between relatives to become “visible” under the influence of cannabis.

**Results:**

Siblings exposed to recent cannabis use resembled their patient-relative more closely in terms of positive schizotypy (urinalysis(+):B = 0.30, P<.001; urinalysis(-):B = 0.10, p<0.001; p-interaction = 0.0135). Similarly, the familial correlation in positive schizotypy between parent and sibling was significantly greater in siblings recently exposed to cannabis (urinalysis(+):B = 0.78, P<.001; urinalysis(-):B = 0.43, p<0.001; p interaction = 0.0017). Results were comparable when using lifetime cannabis frequency of use as exposure instead of recent use. Parental schizotypy did not predict cannabis use in the healthy sibling, nor in the patient. Similarly, parental cannabis use was not associated with level of schizotypy in the sibling, nor with psychotic symptoms in the patient, making gene-environment correlation unlikely.

**Conclusion:**

Familial correlation of psychosis-related experiences varies considerably as a function of exposure to cannabis, confirming the importance of gene-cannabis interaction in shifts of expression of psychosis-related experiences.

## Introduction

Brain development is driven by environmental experience [1], and genetic variation occasions individual differences in environmental response [2], a mechanism referred to as gene-environment interaction [3]. Given strong evidence for both genetic [4] and environmental [5] contribution to psychotic disorder, examination of their joint effects is urgently required [6] but to date remains a neglected area of research [5].

Previous work has shown that cannabis use is associated with schizophrenia [7, 8], and also with the subclinical expression of psychosis in healthy relatives and controls, measured as the psychosis liability phenotype of ‘schizotypy’ [9, 10]. Schizotypy has a heritability of around 50% [11, 12] and the available data suggest a shared genetic vulnerability between the clinical phenotype (psychotic disorder) and the sub-clinical phenotype (schizotypy) [13]. It has therefore been suggested that schizotypy represents an attractive phenotype to study underlying causes of psychotic disorder, with the advantage that it is more prevalent than the clinical phenotype and facilitates research into early changes without the confounds of treatment or illness-related variables [13].

Understanding how familial correlation changes under the influence of environmental stressors is important to grasp the relative contribution of genes, environment and their synergistic effects. If gene-environment interactions play a role in the development of psychotic experiences, familial correlations of psychotic experiences will differ between high-risk and low-risk environments [14]. We wished to examine the familial correlation of psychosis-related experiences between relatives in the same family with at least one affected member, and examine the hypothesis that familial correlations of psychotic experiences are higher in the context of cannabis use. To this end, we used data from an ongoing longitudinal study (Genetic Risk and Outcome in Psychosis; GROUP) in Europe, following patients, siblings and parents pertaining to 1066 families. Conceptually, the level of schizotypy in the healthy sibling was the “response” variable given that this group, given their age, is most likely to develop shifts in expression of psychosis-related experiences under influence of cannabis. Sibling schizotypy thus was modeled as a function of sibling environmental exposure, the corresponding psychosis phenotype in the patient, as well as the interaction between cannabis and patient psychosis phenotype. It was hypothesized that sibling psychosis “response” would display a stronger association with psychosis in the patient with greater level of sibling environmental exposure, following the reasoning that—if gene-environment interactions are important in the development of psychosis—latent underlying genetic vulnerability would become expressed with greater level of environmental exposure, and thus family members would become more comparable in terms of symptom expression under the influence of cannabis. Following the same reasoning, i.e. that familial correlations of psychosis-related phenotypes are stronger in the context of cannabis use, similar analyses were also fitted using the level of positive schizotypy in the parent instead of psychotic symptoms in the patient. Thus, in the parent-sibling analyses, sibling schizotypy was modeled as a function of sibling environmental exposure, parental schizotypy, as well as the interaction between cannabis and parental schizotypy. Positive symptom dimensions of the Structured Interview for Schizotypy-Revised (SIS-R, for siblings and parents) and the Community Assessment of Psychic Experiences (CAPE, for patients) were used as the phenotypes of interest, given evidence that cannabis use most strongly impact the psychotic symptom cluster, rather than negative or general symptom clusters [10, 15].

A crucial aspect in the analyses described above is to distinguish whether familial risk for schizophrenia makes a person more vulnerable to the psychosis-inducing effects of cannabis (gene-environment interaction, or ‘moderation’), or that familial risk for schizophrenia makes a person more likely to start using cannabis (gene-environment correlation, or ‘mediation’). This can be done by analyzing whether schizotypy in the parent predicts cannabis use in the children (patients and siblings). If gene-environment interactions are important in the development of psychotic symptoms, then gene-environment correlation should be negligible or absent.

## Materials and Methods

### Subjects

Full details of the GROUP study have been presented elsewhere [10]. In selected representative geographical areas in the Netherlands and Belgium, patients were identified through representative clinicians working in regional psychotic disorder services, whose caseload was screened for inclusion criteria. Subsequently, a group of patients presenting consecutively at these services either as out-patients or in-patients were recruited for the study. Controls were selected through a system of random mailings to addresses in the catchment areas of the cases.

The full GROUP sample at baseline consisted of 1119 patients with psychotic disorder, 1057 siblings of these 1119 patients, 919 parents of the patients (527 mothers and 392 fathers, 257 families with one parent and 331 families with two parents) and 589 unrelated controls. Inclusion criteria were: (i) age range 16 to 50 years, (ii) clinical diagnosis of a psychotic disorder and (iii) good command of Dutch language. The age range in the siblings was between 14 and 60; 8 siblings were younger than 16 years (0.8%). Families contributed a single patient (206 families, 206 subjects), a single sibling (6 families, 6 subjects), a patient and a sibling (634 families, 1268 subjects), two patients (25 families, 50 subjects), 2 siblings (1 family, 2 subjects), 1 patient and 2 siblings (129 families, 390 subjects), 2 patients and 1 sibling (15 families, 45 subjects), 3 patients (2 families, 6 subjects), 1 patient and 3 siblings (28 families, 112 subjects), 2 patients and 2 siblings (5 families, 20 subjects), 3 patients and 1 sibling (1 family, 4 subjects), 4 patients (1 family, 4 subjects), 1 patient and 4 siblings (8 families, 40 subjects), 2 patients and 3 siblings (2 families, 10 subjects), 3 patients and 2 siblings (2 families, 10 subjects) and 1 patient and 5 siblings (1 family, 6 subjects).

Controls had no first degree relative with a psychotic disorder as established by the Family Interview for Genetic Studies [16] with the control as informant. Diagnosis was based on the Diagnostic and Statistical Manual of Mental Disorder-IV (DSM-IV) criteria [17], assessed with the Comprehensive Assessment of Symptoms and History (CASH) interview [18] or Schedules for Clinical Assessment for Neuropsychiatry (SCAN 2.1)[19]. DSM-IV diagnoses of the patients were: schizophrenia and related disorders (DSM-IV 295.x; n = 940, 84%), other psychotic disorders (DSM-IV 297/298; n = 151, 13%) and psychotic illness in the context of substance-abuse or somatic illness (n = 9; 1%); 9 patients had a missing diagnosis, but fulfilled inclusion criteria, and 10 patients had a final diagnosis of affective psychosis although fulfilling criteria of clinical diagnosis of a psychotic disorder at study entry; these individuals were retained in the sample. In the sibling and control groups, there were respectively 151 (14%) and 60 participants (10%) with a history of a psychiatric disorder other than a psychotic disorder at baseline, the great majority of whom had a mood disorder (DSM-IV 296.x).

### Ethics Statement

The study was approved by the standing ethics committee (Medisch Ethische Toetsingscommissie, UMC Utrecht), and all the subjects gave written informed consent in accordance with the committee's guidelines. This committee waived the need for additional informed consent of parents or supervisors for underaged participants ages 16 and older, given the non-experimental/medical nature of this study.

### Substance Use Measures

Substance use was assessed using the Composite International Diagnostic Interview (CIDI) [20] and through urinalysis. Two different definitions of cannabis exposures were used in the analyses: (i) CIDI cannabis pattern of use during the lifetime period of heaviest use (hereafter: *cannabis frequency use*): none (0), less than weekly (1) weekly (2) and daily (3) and (ii) current cannabis use assessed by urinalysis (hereafter: *cannabis urinalysis*): none (0) and present (1). Urinalysis was carried out as a screen for the presence of cannabis at the national Alcohol- and Drug use ‘Jellinek’c Laboratory. The method used was immunoassays with a cut-off of 50ng/ml. In addition, as an integrity parameter, the creatinine level of every sample was measured. Cannabis urine screening has a detection window up to 30 days, but the detection time has been documented in literature to be even longer (up to three months), depending on level of cannabis use (much shorter in infrequent users) [21]. Given the relatively high cut-off level of 50ng/ml, a conservative detection window of one month can be inferred.

### Familial Psychosis Measures

The shortened Structured Interview for Schizotypy-Revised (SIS-R) [22, 23] was administered to healthy controls, siblings and parents. The SIS-R is a semi-structured interview containing 15 schizotypal symptoms and 4 schizotypal signs, rated on a four-point scale (0 = absent to 3 = severe). Symptoms are defined as verbal responses to standardized questions concerning, for example, magical ideation, illusions, and referential thinking. Signs refer to behaviours that are rated by the interviewer such as, for example, goal directedness of thinking and flatness of affect. Questions and rating procedures are standardized. Guided by previous research [24] and similarly to our previous report [10], positive schizotypy was defined as the mean of the positive schizotypy items (referential thinking, psychotic phenomena, derealisation, magical ideation, illusions, and suspiciousness; range in siblings and parents: 0–2.6). The SIS-R is not ideally suited to assess psychosis in patients due to ceiling effects, however the patients completed the positive dimension of the Community Assessment of Psychic Experiences (CAPE; www.cape42.homestead.com). The CAPE was developed in order to rate self-reports of lifetime psychotic experiences. Psychosis items are modelled on patient experiences as contained in the Present State Examination (PSE)-9 [25] and are scored on a 4-point scale. The CAPE dimension reflecting frequency of positive experiences (20 items) was included for analysis, representing patients’ perceived psychosis load over the lifetime. A total score representing the mean of all items, weighted for partial non-response, was calculated for the positive dimension (hereafter: *CAPE-psychosis*; range in patients in the current sample: 0–2.9).

### Follow-Up

Controls, patients and siblings were eligible for follow-up. Of these, 75% (n = 2074) were assessed at follow-up after three years (controls: 78%, n = 462; sibs: 77%, n = 810; patients: 72%, n = 802). Measures of cannabis use at follow-up reflected use over the interval between baseline and follow-up. Ratings of SIS-R and CAPE at follow-up reflected the period between baseline and follow-up.

### Analyses

Analyses focused on the siblings (n = 1057 at baseline and n = 810 at follow-up). Eight siblings had developed a clinical psychotic disorder at follow-up therefore the follow-up observations of these eight were excluded from the SIS-R analyses. The dependent variable in both the sibling-patient and sibling-parent analyses was the SIS-R positive dimension score (hereafter: *SIS-R psychosis*).

### Sibling-Patient Analysis

For the sibling-patient analyses we calculated the association with patient CAPE psychosis as well as possible moderation thereof by sibling cannabis use (both cannabis urinalysis and cannabis frequency use). Given hierarchical clustering of the data at two levels (clustering of repeated measures within patients and clustering of siblings in the same family) multilevel random regression models, taking into account these two levels, were applied to the data using the *xtmixed* routine in the Stata statistical program, version 12 [26]. Baseline and follow-up measurements were thus combined into one analysis, taking into account within-person clustering of repeated measurements. Post-hoc comparisons examined the degree of familial correlation at different levels of cannabis use using the “Margins” command in Stata. In order to reliably examine patient-sibling correlation it is important that the phenotype in the sibling resembles the patient phenotype as closely as possible. Since cannabis most strongly impacts on the positive psychotic symptoms of schizophrenia and other psychotic disorders [10], this was the phenotype in the patient we were most interested in (as measured by the positive subscale of the CAPE). In order to obtain a measure that most closely resembles this phenotype in the sibling, we used a set of SIS-R items capturing subclinical psychosis, as applied previously [10]. As a sensitivity analysis, we repeated the analyses using total SIS-R rather than SIS-R psychosis. Associations were expressed as the regression coefficient (B) from the multilevel random regression model. All analyses were *a priori* adjusted for age, sex and ethnic group (White versus Non-white) given that these variables are associated with the incidence of psychosis [27, 28]. Analyses were done without, as well as with additional adjustment for lifetime cannabis use, in order to examine to what degree familial correlation in psychosis phenotypes was mediated by familial correlation in cannabis use, models were also adjusted for parental and patient *cannabis lifetime use*.

### Sibling-Parent Analysis

For the sibling-parent analyses, we similarly calculated the association between sibling SIS-R psychosis as dependent variable and parental SIS-R psychosis as well as possible moderation thereof by sibling cannabis use (both cannabis urinalysis and cannabis frequency use) as independent variables. Again, all analyses were *a priori* adjusted for age, sex and ethnic group. Analyses were done without, as well as with additional adjustment for lifetime cannabis use as described above. If a sibling had more than one participating parent, the mean SIS-R psychosis of both parents was used to calculate the association with sibling SIS-R psychosis, since it is unknown whether the familial vulnerability comes from the maternal or paternal side, or both. The same procedure was applied to CAPE psychosis when a family contributed more than one patient. There were 257 families with one parent and 331 families with two parents. Since the average of both parents was used for the families with two parents, 588 sibling-parent pairs were available for the analysis.

### Moderation versus Mediation

In order to determine to what degree results in siblings, parents and patients reflect moderation (environmental exposure induces expression of psychosis in those with higher levels of familial risk) rather than mediation (familial risk induces environmental exposure), we examined whether schizotypy in the parent predicts cannabis use in the children (patients and siblings), controlling for cannabis use in the parent, age, sex and ethnicity. Similarly, we examined whether parental cannabis use was associated with schizotypy in the sibling, and psychosis in the patient, controlling for parental schizotypy, age, sex and ethnicity. Absence of significant associations would indicate absence of gene-environment correlation or mediation.

## Results

### Sample

The sample consisted of 589 controls and 1066 families who contributed 1057 siblings and 1119 patients. The median illness duration of patients at baseline was 3.2 years (interquartile range: 1.2–6.0).

Siblings had cannabis exposure and psychosis-related experiences scores that were intermediate to those of parents (lowest) and patients (highest) (Table 1). *Cannabis lifetime use* in the siblings was associated with *cannabis lifetime use* in the parents (OR = 3.3, 95% CI: 2.0, 5.4) and the patients (OR = 4.9, 95% CI: 3.1, 7.7). As expected, the a priori confounders age (B = -.006, SE .001, p<.0001), sex (B = .058, SE .021, p = .006) and ethnicity (B = .091, SE .03, p = .003) were all significantly associated with sibling SIS-R psychosis.

**Table 1:** Exposure, outcome and a priori confounding variables in controls, siblings and patients at baseline (lifetime assessment) and follow-up (interval assessment).

	*Controls*		*Parents*		*Siblings*		*Patients*	
	*Baseline (N = 589)*	*Follow-up (N = 458)*	*Baseline (N = 919)*	*Follow-up (-)*	*Baseline (N = 1057)*	*Follow-up (N = 802)*	*Baseline (N = 1119)*	*Follow-up (N = 802)*
Age	30.3 (10.6)	34.2 (10.6)	54.8 (6.8)	—	27.8 (8.3)	30.5 (7.9)	27.6 (8.0)	30.1 (7.2)
Male sex	269 (46%)	202 (44%)	392 (43%)	—	482 (46%)	355 (44%)	853 (76%)	615 (77%)
White ethnic group	530 (90%)	421 (91%)	811 (88%)	—	877 (83%)	698 (87%)	857 (77%)	657 (82%)
Cannabis frequency use* (n, %)	N = 584	N = 455	N = 915	—	N = 1042	N = 791	N = 1092	N = 788
None	423 (72%)	392 (86%)	820 (90%)	—	643 (62%)	657 (83%)	403 (37%)	558 (71%)
< weekly	79 (14%)	36 (8%)	43 (5%)	—	146 (14%)	61 (8%)	94 (9%)	66 (8%)
weekly	39 (7%)	10 (2%)	28 (3%)	—	101 (10%)	29 (4%)	120 (11%)	41 (5%)
daily	43 (7%)	17 (4%)	24 (3%)	—	152 (15%)	44 (6%)	475 (44%)	123 (16%)
Cannabis urinalysis	N = 560	N = 455	—	—	N = 950	N = 791	N = 974	N = 791
	27 (5%)	14 (3%)	—	—	74 (8%)	47 (6%)	158 (16%)	87 (11%)
Cannabis lifetime use†	N = 584	N = 455	N = 915	—	N = 1042	N = 791	N = 1092	N = 788
	165 (28%)	135 (30%)	100 (11%)	—	409 (39%)	328 (41%)	710 (65%)	514 (65%)
SIS-R positive (SD, range)	N = 581	N = 451	N = 912	—	N = 1038	N = 785		
	0.31 (0.35; 0–2.1)	0.28 (0.31; 0–2.3)	0.27 (0.33; 0–2.4)	—	0.38 (0.42; 0–2.6)	0.31 (0.34; 0–2.6)	NA	NA
CAPE positive (SD, range)	N = 562	N = 445	N = 777	—	N = 919	N = 770	N = 877	N = 743
	0.19 (0.18; 0–1.0)	0.09 (0.12; 0–0.9)	0.14 (0.14; 0–1.5)	—	0.21 (0.20; 0–1.2)	0.11 (0.14; 0–1.5)	0.67 (0.49; 0–2.9)	0.52 (0.50; 0–2.9)

*CIDI cannabis pattern of use during the lifetime period of heaviest use; indicates lifetime frequency of use (at baseline) and frequency of use between baseline and follow-up assessment (at follow-up), respectively

^†^Any use of cannabis during the lifetime

—not included in follow-up

NA: not applicable

### Sibling-Patient Psychosis Association as a Function of Sibling Cannabis Use

**Fig 1:**
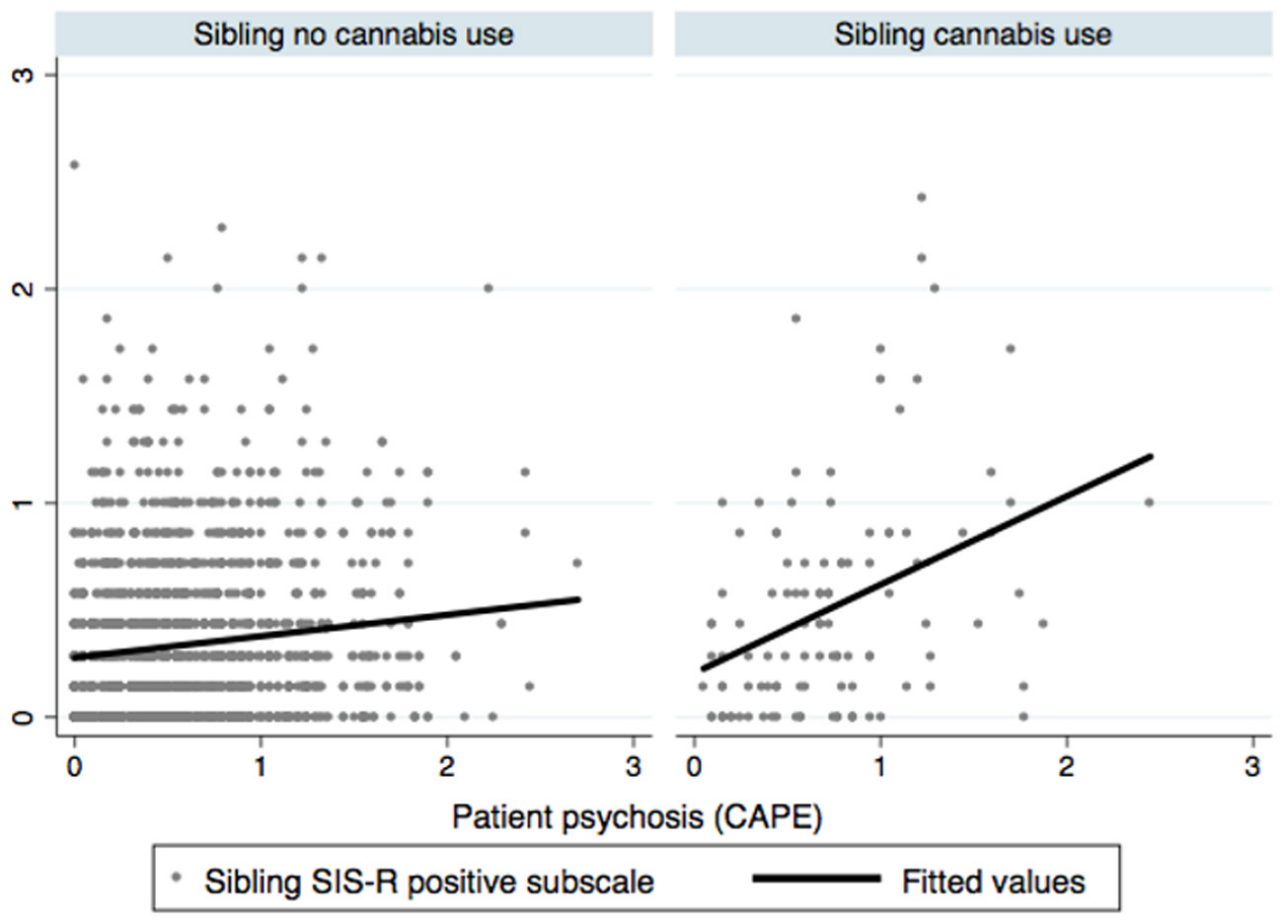
Correlation between patient psychotic symptoms (CAPE) and sibling positive schizotypy depending on recent exposure to cannabis in the sibling. Legend: The correlation between sibling SIS-R psychosis (Y-axis) and psychosis in the patient (X-axis). Left panel: correlation in the absence of recent cannabis use in the sibling. Right panel: correlation in the presence of recent cannabis use in the sibling.

Mean levels of patient *CAPE-psychosis* and sibling *SIS-R psychosis* as a function of *cannabis frequency use* and *cannabis urinalysis* are depicted in Tables 2 and 3. The correlation between sibling and patient psychosis phenotypes (n = 978 pairs, n = 1723 observations) was greater with greater levels of cannabis use in the sibling. In the model of sibling *SIS-R psychosis*, adjusted for age, sex and ethnic group, there was a significant positive interaction between sibling *cannabis frequency use* and patient *CAPE-psychosis* (χ^2^ = 9.82, df = 3, p = 0.0202), which was not affected by additional adjustment for patient *cannabis lifetime use* (χ^2^ = 9.86, df = 3, p = 0.0198; Table 4). Similarly, there was a significant positive interaction between sibling *cannabis urinalysis* and patient *CAPE*-*psychosis* in the model adjusted for age, sex and ethnic group (χ^2^ = 9.01, df = 1, p = 0.0027), which was unaltered after additional adjustment for patient *cannabis lifetime use* (χ^2^ = 8.87, df = 1, p = 0.0029; Table 4 and Fig 1). Similar results were found when using sibling total SIS-R score instead of SIS-R psychosis as the dependent variable for the interaction with sibling *cannabis frequency use* (χ^2^ = 8.13, df = 3, p = 0.0435; after additional adjustment for patient *cannabis lifetime use*: χ^2^ = 8.12, df = 3, p = 0.0437). The interaction with sibling *cannabis urinalysis* was no longer statistically significant at conventional alpha, however (χ^2^ = 2.87, df = 1, p = 0.0905; after additional adjustment for patient *cannabis lifetime use*: χ^2^ = 2.78, df = 1, p = 0.0955).

**Table 2:** Mean level of familial psychosis phenotypes by level of sibling *cannabis frequency use*, by group and follow-up period.

	SIS-R psychosis						CAPE psychosis			
	Siblings				Parents		Patients			
Sibling cannabis frequency*	Baseline	n	Follow-up	n	Baseline	n	Baseline	n	Follow-up	n
None	0.35	639	0.30	651	0.25	403	0.61	604	0.62	631
Less than weekly	0.35	146	0.34	59	0.23	97	0.62	139	0.54	59
Weekly	0.42	100	0.38	29	0.30	75	0.66	96	0.80	28
Daily	0.54	149	0.43	44	0.36	96	0.67	138	0.70	42

*CIDI cannabis pattern of use during the lifetime period of heaviest use

**Table 3:** Mean level of familial psychosis phenotypes by level of sibling *cannabis urinalysis*, by group and follow-up period.

	SIS-R psychosis						CAPE psychosis			
	Siblings				Parents		Patients			
Sibling cannabis urinalysis	Baseline	n	Follow-up	n	Baseline	n	Baseline	n	Follow-up	n
Negative	0.37	866	0.30	736	0.26	568	0.61	868	0.62	715
Positive	0.56	72	0.45	47	0.41	39	0.77	63	0.72	45

**Table 4:** Sibling-patient SIS-R/CAPE psychosis association as a function of sibling cannabis use, assessed as *cannabis frequency use* (top) and *cannabis urinalysis* (bottom). Depicted are models of *SIS-R psychosis* in the sibling as dependent variable and sibling cannabis use, patient *CAPE-psychosis* and their interaction as independent variables, adjusted for age, sex, ethnic group and patient *cannabis lifetime use*. Stratified associations were derived from the model by linear combination of effects.

*Sibling cannabis frequency* *	*Stratified sibling-patient psychosis association* †	*95% CI*	
none	0.08	0.02	0.13
less	0.15	0.03	0.27
weekly	0.12	-0.01	0.25
daily	0.27	0.16	0.39
*Sibling cannabis urinalysis*	*Stratified sibling-patient psychosis association* †	*95% CI*	
negative	0.09	0.04	0.15
positive	0.34	0.18	0.50

*CIDI cannabis pattern of use during the lifetime period of heaviest use

^†^ Associations represent the regression coefficient (B) from the multilevel random regression model

### Sibling-Parent Psychosis Association as a Function of Sibling Cannabis Use

Mean levels of sibling and parental *SIS-R psychosis* as a function of *cannabis frequency use* and *cannabis urinalysis* are depicted in Tables 2 and 3. The correlation between sibling and parental *SIS-R psychosis* (n = 669 pairs, n = 1222 observations) was greater with greater levels of cannabis use in the sibling (Table 4). In the model of sibling *SIS-R psychosis*, adjusted for age, sex and ethnic group, there was a significant positive interaction between *cannabis frequency use* and parental *SIS-R psychosis* (χ^2^ = 10.8, df = 3, p = 0.0128; Table 5), which was not attenuated after additional adjustment for parental *cannabis lifetime use* (χ^2^ = 11.0, df = 3, p = 0.0117). Similarly, there was a significant positive interaction between *cannabis urinalysis* and parental *SIS-R psychosis* in the model adjusted for age, sex and ethnic group (χ^2^ = 8.33, df = 1, p = 0.0039), which remained similar after additional adjustment for parental *cannabis lifetime use* (χ^2^ = 8.32, df = 1, p = 0.0039; Table 5). When examining the familial correlation using total SIS-R score instead of SIS-R psychosis, it was found that this correlation was no longer moderated by sibling *cannabis frequency use* (χ^2^ = 5.50, df = 3, p = 0.1387; after additional adjustment for patient *cannabis lifetime use*: χ^2^ = 5.65, df = 3, p = 0.130) or sibling *cannabis urinalysis* (χ^2^ = 1.50, df = 1, p = 0.221; after additional adjustment for patient *cannabis lifetime use*: χ^2^ = 1.57, df = 1, p = 0.210).

**Table 5:** Sibling-parent SIS-R psychosis association as a function of sibling cannabis use, assessed as *cannabis frequency use* (top) and *cannabis urinalysis* (bottom). Depicted are models of SIS-R psychosis in the sibling as dependent variable and sibling cannabis use, parental SIS-R psychosis and their interaction as independent variables, adjusted for age, sex, ethnic group and parental *cannabis lifetime use*. Stratified associations were derived from the model by linear combination of effects.

*Sibling cannabis frequency* *	*Stratified sibling-parent psychosis association* †	*95% CI*	
none	0.38	0.29	0.47
less	0.39	0.21	0.58
weekly	0.56	0.38	0.73
daily	0.65	0.49	0.80
*Sibling cannabis urinalysis*	*Stratified sibling-parent psychosis association* †	*95% CI*	
negative	0.44	0.36	0.52
positive	0.77	0.55	0.99

*CIDI cannabis pattern of use during the lifetime period of heaviest use

^†^ Associations represent the regression coefficient (B) from the multilevel random regression model

### Moderation versus Mediation

Parental schizotypy was not associated with cannabis use in the healthy sibling (B = .01, SE .03, p = .80), nor with cannabis use in the patient (B = .005, SE .03, p = .86). Similarly, parental cannabis use was not associated with level of schizotypy in the sibling (B = -.04, SE .04, p = .32), nor with psychotic symptoms in the patient (B = -.10, SE .06, p = .09).

## Discussion

The familial correlation in psychosis-related experiences between individuals at familial risk for psychotic disorder was moderated by exposure to cannabis use. Results were consistent across correlations in psychosis-related experiences between healthy relative-pairs (sibling-parent pairs) as well as liability-illness correlations in sibling-patient pairs. Analysis showed that the pattern of results was not reducible to familial correlation of cannabis *per se* and our analyses did not provide evidence for mediation as an alternative explanation, suggesting that the use of cannabis is not a byproduct of underlying psychosis liability (mediation), but rather an environmental factor acting synergistically with familial liability to produce symptoms of psychosis (moderation). It must be noted that these results were less consistent when analyzing total SIS-R score instead of the positive psychotic items of the SIS-R, confirming that cannabis use particularly impacts on the positive psychotic symptoms [10, 15]. Familial correlation of these positive psychosis-related experiences was 2 to 3 times greater in the context of cannabis use, suggesting that a considerable portion of phenotypic variation in psychosis-related experiences is explained by gene-environment interactions with cannabis.

Previous work in the same sample showed that unaffected siblings of patients with schizophrenia displayed much greater sensitivity to the effects of cannabis use than controls, as well as resembling their patient relative more closely in the positive psychotic dimension of the CAPE compared to non-exposed siblings [10]. Compared to this previous report, the present paper adds further weight to the notion of interaction between familial risk and cannabis use, as it is based on a substantially larger dataset, which includes baseline and 3-year follow-up measurements (n = 978 pairs, n = 1723 observations), as well as a novel analysis of 669 sibling-parent pairs (n = 1222 observations).

Strengths of the study include the within-study sibling-parent and sibling-patient replication of environmental moderation of familial phenotypic resemblance, the large sample size and the cannabis exposure assessment by urinalysis. In addition, both the sibling-parent and the sibling-patient correlational analysis has the important advantage of automatic control for a range of confounders that may affect case-control comparisons in unrelated subjects, given the fact that relatives share a range of demographic factors and life circumstances that may impact on mental health and substance use. The disadvantage of the familial design in this context is that familial correlations are influenced by familial sharing of environmental exposures in addition to shared genes, however the analyses suggest that results were not reducible to familial clustering of environmental exposures *per se*.

### Limitations

The results should be interpreted in the light of the following limitations. As schizotypy is not a measure that can be reliably assessed in patients with psychotic disorder (ceiling effects), the psychosis measure for the patients, used in the sibling-patient analysis, was self-reported psychotic experiences using the CAPE. It could be argued that the CAPE does not measure the same phenotype as the SIS-R in the siblings. However, for the purpose of the cross-sibling association analyses, phenotypic similarity is not required; what is required is that the measure in both groups taps into the same underlying liability, at the level of behavioral expression of liability in the siblings, and at the level of illness-related symptoms in the patients. As previous work has shown strong concurrent validity of the CAPE with schizotypy in healthy controls [29], and concurrent validity with positive psychotic symptoms in patients, regardless of level of insight [30], the SIS-R-CAPE cross-sib association can be interpreted as the sibling correlation in the level of psychosis-related experiences. Recent evidence from genome-wide studies suggests considerable genetic heterogeneity associated with schizophrenia [4], which is impossible to take into account in the present analysis at the level of familial risk for non-affective psychosis. However, as analyses focused on pairs of relatives, who by definition pertain to similar genetic strata, genetic heterogeneity is unlikely to explain the results presented here. Lastly, SIS-R psychosis, as used in this and previous studies, was not originally described in the report by Vollema and Ormel as a separate subscale [23]. Nevertheless, Vollema and Ormel note in their paper that “most schizotypal symptoms and signs are reliable to assess, suggesting that dimensions of schizotypy, which are framed by specific symptoms and signs, can be reliably assessed too” [23]. Therefore, we believe that the use of the positive psychotic items in the SIS-R is not only statistically most appropriate in order to sensibly analyze correlations of positive psychotic dimensions among sibling-patient pairs, but its use as a separate dimension of schizotypy is also conceptually and psychometrically valid.

## Supporting Information

S1 Datafile Datafile with the underlying data. (DTA)

## References

[1] Wiesel TN, Hubel DH. Effects of Visual Deprivation on Morphology and Physiology of Cells in the Cats Lateral Geniculate Body. J Neurophysiol. 1963;26:978–93. Epub 1963/11/01. . 14084170 10.1152/jn.1963.26.6.978

[2] Tyron RC. Genetic differences in maze-learning ability in rats. Yearbook of the National Society for the Study of Education. 1940;39:111–9.

[3] Khoury MJ, Beaty TH, Cohen BH. Genetic Epidemiology. Oxford: Oxford University Press; 1993.

[4] O'Tuathaigh CM, Hryniewiecka M, Behan A, Tighe O, Coughlan C, Desbonnet L, et al Chronic adolescent exposure to delta-9-tetrahydrocannabinol in COMT mutant mice: impact on psychosis-related and other phenotypes. Neuropsychopharmacology. 2010;35(11):2262–73. 10.1038/npp.2010.100 20631688 PMC3055315

[5] van Os J, Kenis G, Rutten BP. The environment and schizophrenia. Nature. 2010;468(7321):203–12. Epub 2010/11/12. nature09563 [pii] 10.1038/nature09563 . 21068828

[6] Demjaha A, MacCabe JH, Murray RM. How genes and environmental factors determine the different neurodevelopmental trajectories of schizophrenia and bipolar disorder. Schizophr Bull. 2012;38(2):209–14. Epub 2011/08/23. sbr100 [pii] 10.1093/schbul/sbr100 21857009 PMC3283142

[7] Matheson SL, Shepherd AM, Laurens KR, Carr VJ. A systematic meta-review grading the evidence for non-genetic risk factors and putative antecedents of schizophrenia. Schizophr Res. 2011;133(1–3):133–42. Epub 2011/10/18. S0920-9964(11)00499-3 [pii] 10.1016/j.schres.2011.09.020 . 21999904

[8] Varese F, Smeets F, Drukker M, Lieverse R, Lataster T, Viechtbauer W, et al Childhood Adversities Increase the Risk of Psychosis: A Meta-analysis of Patient-Control, Prospective- and Cross-sectional Cohort Studies. Schizophr Bull. 2012;Epub ahead of print. Epub 2012/03/31. sbs050 [pii] 10.1093/schbul/sbs050 . 22461484 PMC3406538

[9] Heins M, Simons C, Lataster T, Pfeifer S, Versmissen D, Lardinois M, et al Childhood trauma and psychosis: a case-control and case-sibling comparison across different levels of genetic liability, psychopathology, and type of trauma. Am J Psychiatry. 2011;168(12):1286–94. Epub 2011/10/01. 10.1176/appi.ajp.2011.10101531 [pii]. . 21955935

[10] Genetic Risk and Outcome of Psychosis (GROUP) Investigators. Evidence that Familial Liability for Psychosis is Expressed as Differential Sensitivity to Cannabis: an Analysis of Patient-Sibling and Sibling-Control Pairs. Arch Gen Psychiatry. 2011;68(2):138–47. 10.1001/archgenpsychiatry.2010.132 20921112

[11] Franke TF. PI3K/Akt: getting it right matters. Oncogene. 2008;27:6473–88. 10.1038/onc.2008.313 18955974

[12] Freyberg Z, Ferrando SJ, Javitch JA. Roles of the Akt/GSK-3 and Wnt signaling pathways in schizophrenia and antipsychotic drug action. Am J Psychiatry. 2010;167(4):388–96. 10.1176/appi.ajp.2009.08121873 19917593 PMC3245866

[13] Kelleher I, Cannon M. Psychotic-like experiences in the general population: characterizing a high-risk group for psychosis. Psychol Med. 2010;19:1–6. 10.1017/S0033291710001005 20624328

[14] Kendler KS, Eaves LJ. Models for the joint effect of genotype and environment on liability to psychiatric illness. Am J Psychiatry. 1986;143(3):279–89. 3953861 10.1176/ajp.143.3.279

[15] van Winkel R, Kuepper R. Epidemiological, neurobiological, and genetic clues to the mechanisms linking cannabis use to risk for nonaffective psychosis. Annu Rev Clin Psychol. 2014;10:767–91. 10.1146/annurev-clinpsy-032813-153631 24471373

[16] NIMH.Genetics.Initiative. Family Interview for Genetic Studies (FIGS). Rockville, Md: National Institute of Mental Health; 1992.

[17] Association AP. Diagnostic and Statistical Manual of Mental Disorders, 4th ed., Text Revision. Washington, D.C.: American Psychiatric Association; 2000.

[18] Andreasen NC, Flaum M, Arndt S. The Comprehensive Assessment of Symptoms and History (CASH). An instrument for assessing diagnosis and psychopathology. Arch Gen Psychiatry. 1992;49(8):615–23. 1637251 10.1001/archpsyc.1992.01820080023004

[19] Wing JK, Babor T, Brugha T, Burke J, Cooper JE, Giel R, et al SCAN. Schedules for Clinical Assessment in Neuropsychiatry. Arch Gen Psychiatry. 1990;47(6):589–93. 2190539 10.1001/archpsyc.1990.01810180089012

[20] World Health Organisation. Composite International Diagnostic Interview (CIDI) Version 1.0. Geneva: World Health Organisation; 1990.

[21] Musshoff F, Madea B. Review of biologic matrices (urine, blood, hair) as indicators of recent or ongoing cannabis use. Ther Drug Monit. 2006;28(2):155–63. Epub 2006/04/22. 10.1097/01.ftd.0000197091.07807.22 00007691-200604000-00002 [pii]. . 16628124

[22] Kendler KS, Lieberman JA, Walsh D. The Structured Interview for Schizotypy (SIS): a preliminary report. Schizophr Bull. 1989;15(4):559–71. . 2623438 10.1093/schbul/15.4.559

[23] Vollema MG, Ormel J. The reliability of the structured interview for schizotypy-revised. Schizophr Bull. 2000;26(3):619–29. Epub 2000/09/19. . 10993402 10.1093/oxfordjournals.schbul.a033482

[24] Hanssen M, Krabbendam L, Vollema M, Delespaul P, Van Os J. Evidence for instrument and family-specific variation of subclinical psychosis dimensions in the general population. J Abnorm Psychol. 2006;115(1):5–14. . 16492091 10.1037/0021-843X.115.1.5

[25] Wing JK, Cooper JE, Sartorius N. The measurement and classification of psychiatric symptoms. London: Cambridge University Press; 1974.

[26] StataCorp. STATA Statistical Software: Release 12. Texas: College Station; 2011.

[27] Bromet EJ, Kotov R, Fochtmann LJ, Carlson GA, Tanenberg-Karant M, Ruggero C, et al Diagnostic shifts during the decade following first admission for psychosis. Am J Psychiatry. 2011. 10.1176/appi.ajp.2011.11010048 PMC3589618 21676994

[28] Wang Y, Localio R, Rebbeck TR. Evaluating bias due to population stratification in epidemiological studies of gene-gene or gene-environment interactions. Cancer Epidemiol Biomarkers Prev. 2006;15:124–32. 16434597 10.1158/1055-9965.EPI-05-0304

[29] Konings M, Bak M, Hanssen M, van Os J, Krabbendam L. Validity and reliability of the CAPE: a self-report instrument for the measurement of psychotic experiences in the general population. Acta Psychiatr Scand. 2006;114(1):55–61. . 16774662 10.1111/j.1600-0447.2005.00741.x

[30] Liraud F, Droulout T, Parrot M, Verdoux H. Agreement between self-rated and clinically assessed symptoms in subjects with psychosis. J Nerv Ment Dis. 2004;192(5):352–6. Epub 2004/05/06. 00005053-200405000-00003 [pii]. . 15126889 10.1097/01.nmd.000126702.30745.1d

